# Supplementary material for: Feature selection enhances peptide binding predictions for TCR-specific interactions
Source: Front Immunol. 2025 Jan 23;15:1510435. doi: 10.3389/fimmu.2024.1510435 (PMC11799297; doi:10.3389/fimmu.2024.1510435)
Supplement: Supplementary file 1 [file DataSheet1.pdf]

Feature Selection Enhances Peptide Binding Predictions for TCR-Specific Interactions

Hamid Teimouri [1,2,†], Zahra S. Ghoreyshi [2,3,†], Anatoly B. Kolomeisky [1,2,4], and Jason T. George [2,3,5,6]

[1]Department of Chemistry, Rice University, Houston, TX, USA
[2]Center for Theoretical Biological Physics, Rice University, Houston, TX, USA
[3]Department of Biomedical Engineering, Texas A&M University, College Station, TX, USA
[4]Department of Chemical and Biomolecular Engineering, Rice University, Houston, TX, USA
[5]Department of Hematopoietic Biology and Malignancy, MD Anderson Cancer Center, Houston, TX, USA
[6]Department of Translational Medical Sciences, Texas A&M Health Science Center, Houston, TX, USA
[†]These authors contributed equally to this work and share first authorship

1 SUPPLEMENTARY FIGURES

In this supporting information, we present additional figures that are discussed in the main text.

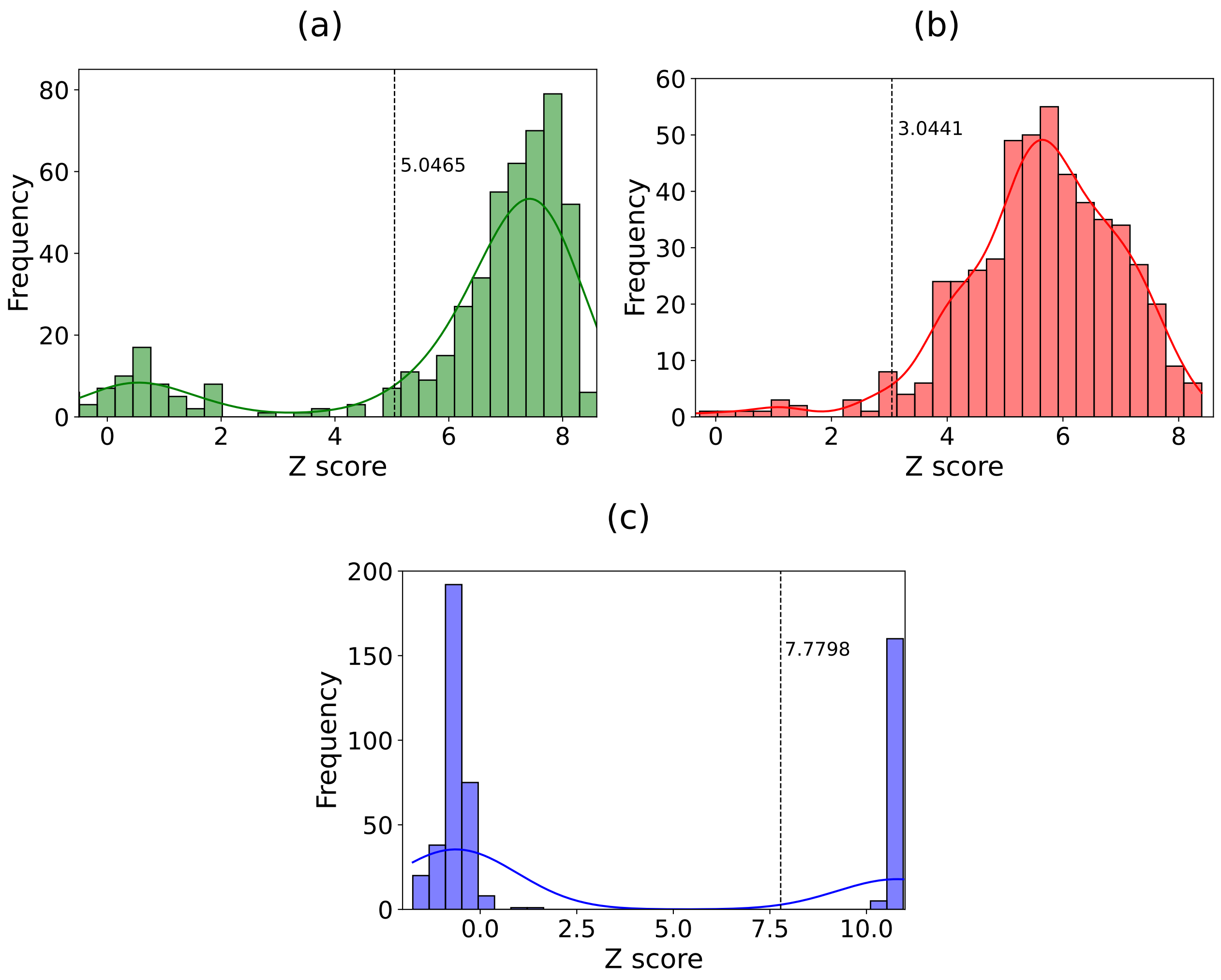

Figure S1: Calculation of threshold value suing RACER model for peptide libraries associate with (a) 2B4 TCR , (b) 226 TCR , (c) 5cc7 TCR.

1.1 Histograms for Calculating Thresholds

Corresponding histograms for calculating post selection abundance threshold, which separates strong binders from weak binders, are presented in Fig. S7.

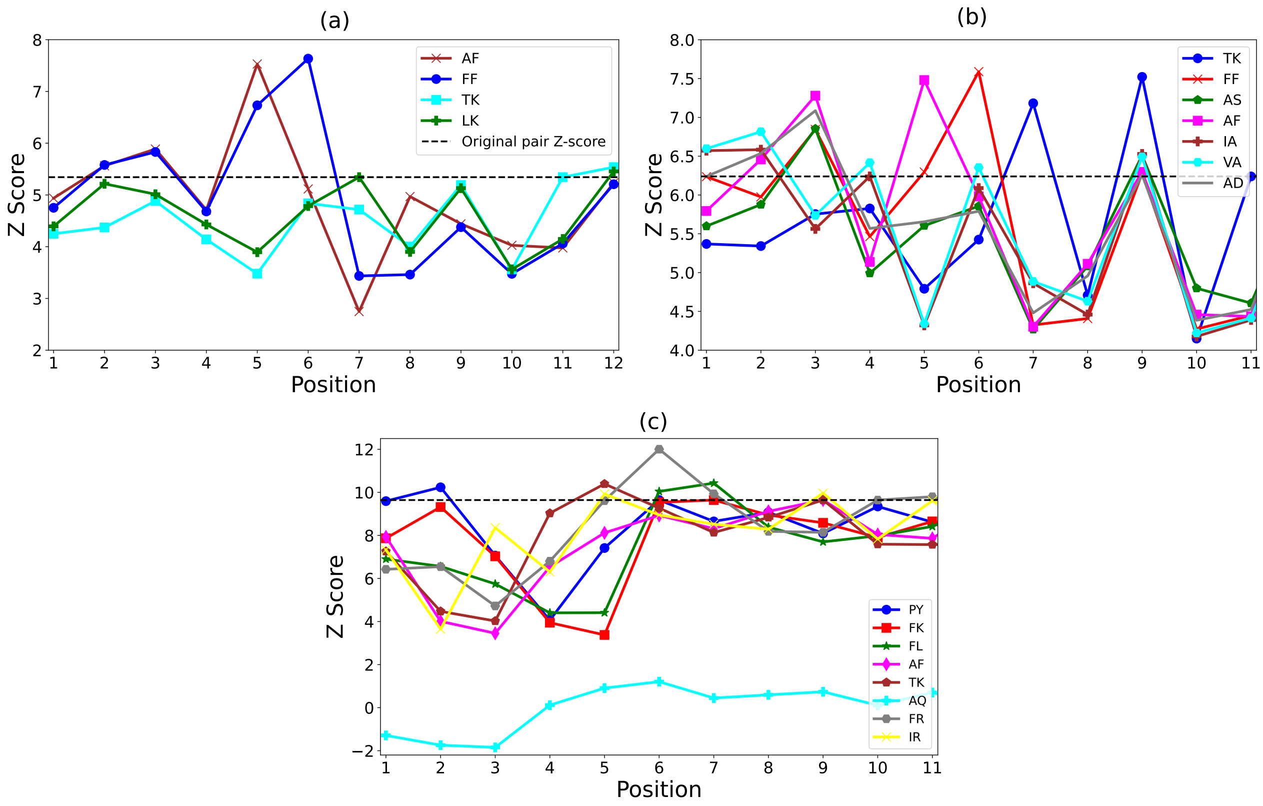

Figure S2: RACER model predictions for the selected dipeptide compositions for (a) 2B4, (b) 226, (c) 5cc7.

1.2 Dipeptides from RACER model

The corresponding position curves, calculated by the RACER model for dipeptide feature selected by LASSO, are presented in Fig. S2.

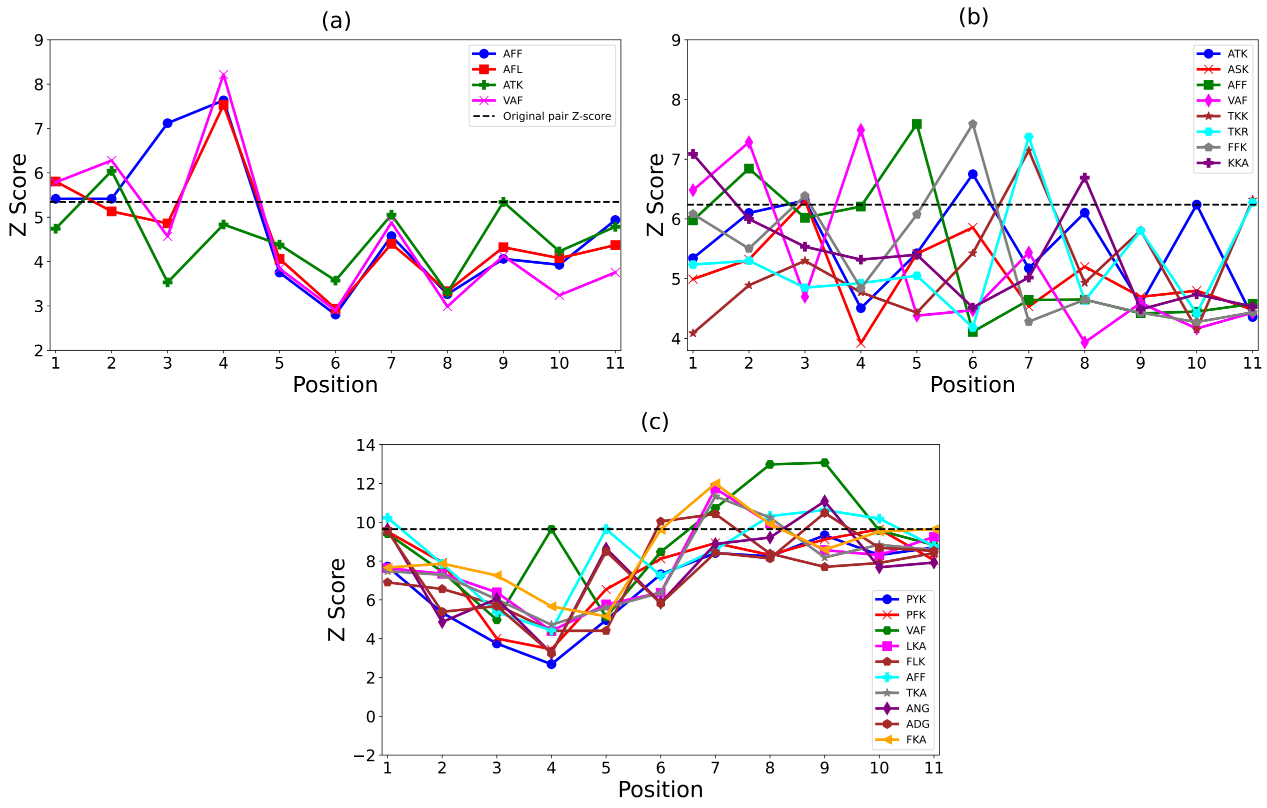

Figure S3: RACER model predictions for the selected tripeptide compositions for (a) 2B4, (b) 226, (c) 5cc7.

1.3 Tripeptide from RACER model

Fig. S3 displays the position curves for tripeptide features selected by LASSO, calculated using the RACER model.

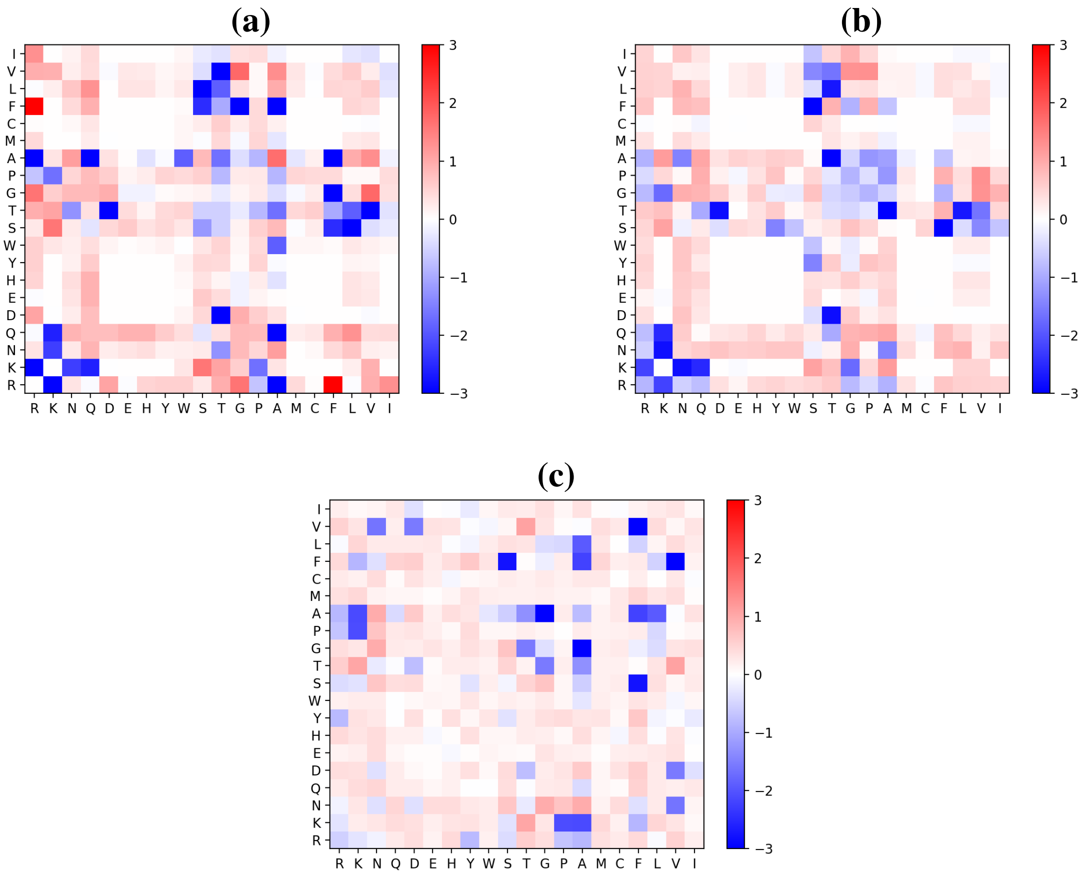

Figure S4: The residue-based interaction strength, represented by the energy matrix, determined by RACER with a maximum distance of $r_{\text{max}} = 8.5$Åfor TCR (a) 2B4, (b) 226, and (c) 5cc7.

1.4 Analysis of TCR-Peptide contact maps and energy matrices

In this study, we present RACER-driven energy matrices and corresponding contact maps for various TCR-peptide complexes, all generated using a maximum distance cutoff of 8.5Å (Fig. S4 and S5).

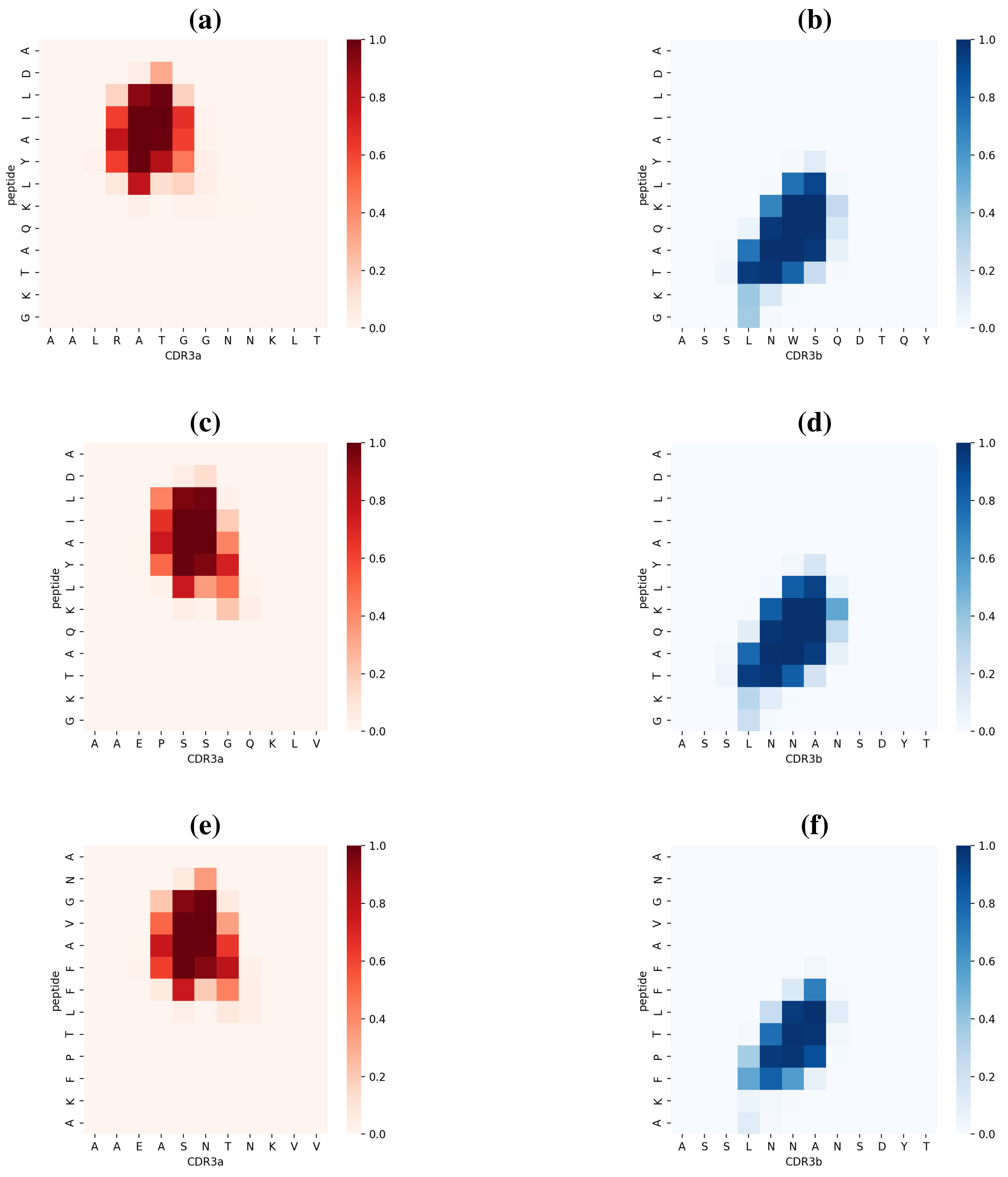

Figure S5: Contact maps illustrating the peptide-TCR interactions for different TCR-peptide complexes, generated using a maximum distance ($r_{\text{max}} = 8.5\text{Å}$): **(a)** 2B4 CDR3$\alpha$, **(b)** 2B4 CDR3$\beta$, **(c)** 226 CDR3$\alpha$, **(d)** 226 CDR3$\beta$, **(e)** 5cc7 CDR3$\alpha$, and **(f)** 5cc7 CDR3$\beta$.

Table S1. Description of selected *propy* features related to Figs 3(a) and Fig 4(a) in the main text.

Feature Acronym	Feature Description
PolarityD2075	The fraction of the entire sequence, where 75% of the residues of group 2 (polarity values $8.0 - 9.2$) are contained.
GearyAuto_Hydrophobicity5	Geary's autocorrelation function of hydrophobicity for amino acids that are 5 positions apart.
QSOSW12	Quasi-sequence order
QSOgrant5	Quasi-order-coupling number
NormalizedVDWVC2	Global percent composition of residues with normalized van der Waals volume in range $2.95 - 94.0$.

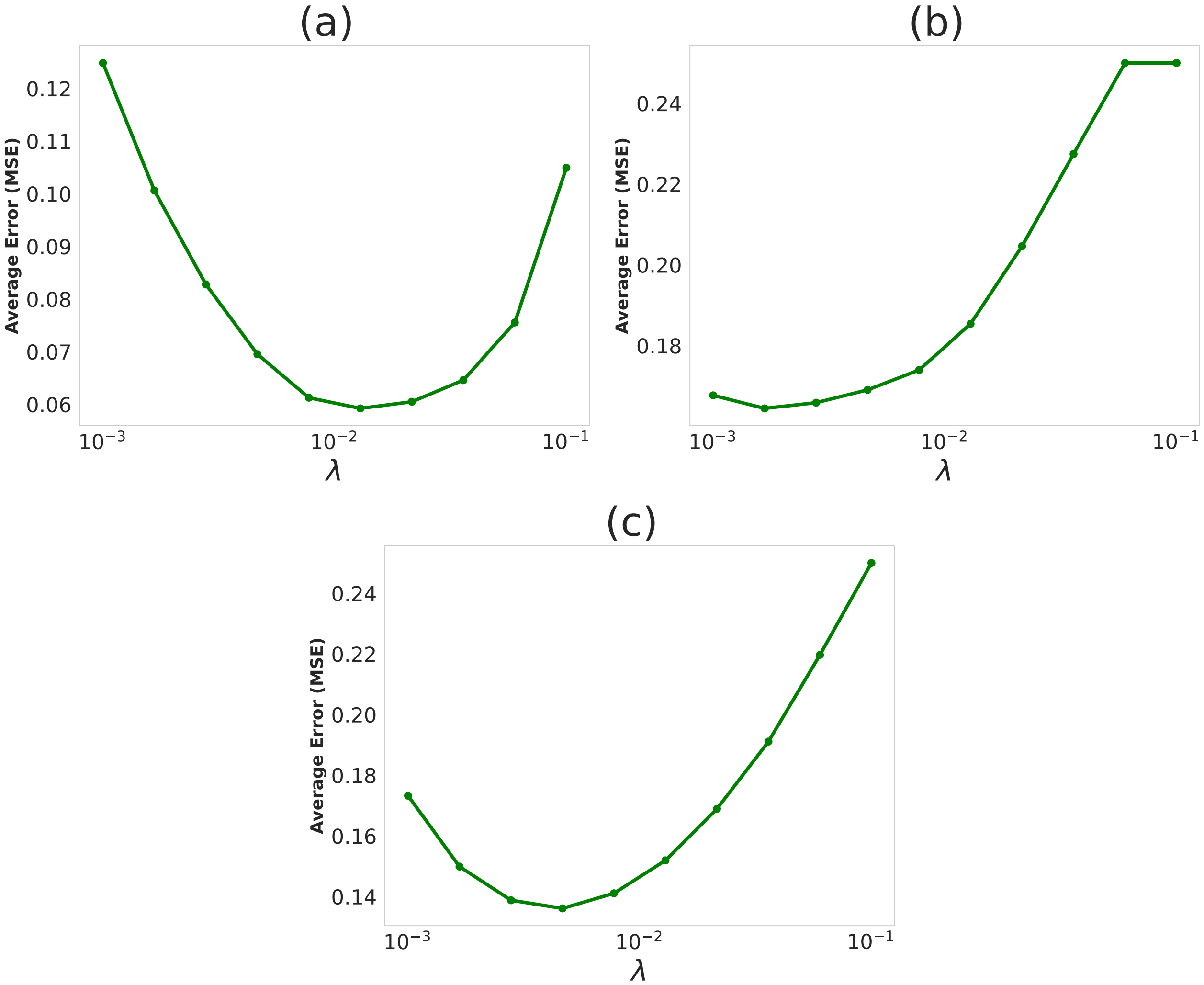

Figure S6: Average mean square error (MSE) as a function of the hyperparameter (λ) for LASSO-based feature selection. The analysis utilized *propy* features extracted from peptides associated with (a) 2B4 TCR, (b) 226 TCR, (c) 5cc7 TCR.

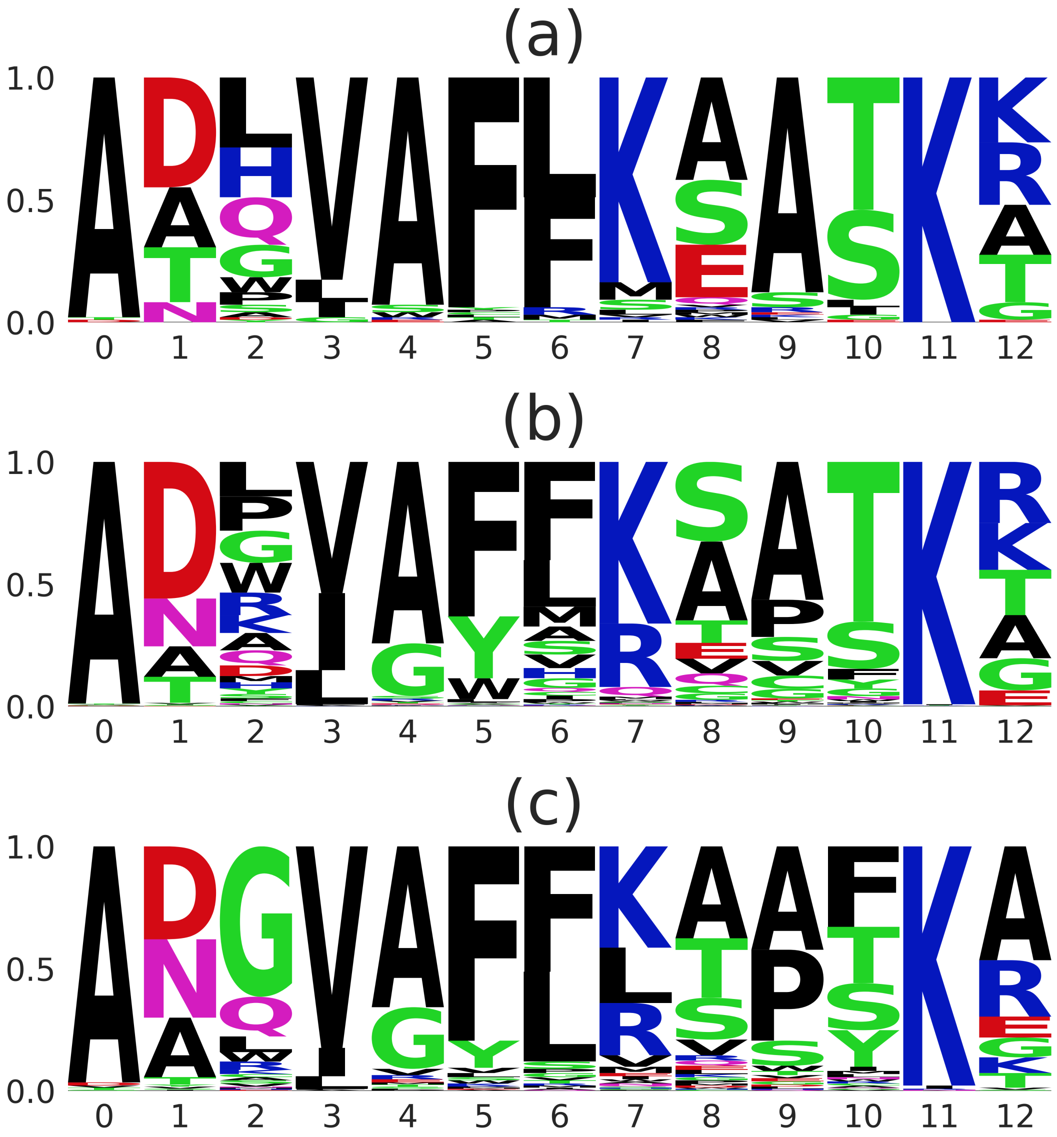

Figure S7: Sequence logos representing strong binder peptides targeting (a) 2B4 TCR, (b) 226 TCR, (c) 5cc7 TCR.
